# In search of cytotoxic selectivity on cancer cells with biogenically synthesized Ag/AgCl nanoparticles

**DOI:** 10.3762/bjnano.13.124

**Published:** 2022-12-13

**Authors:** Mitzi J Ramírez-Hernández, Mario Valera-Zaragoza, Omar Viñas-Bravo, Ariana A Huerta-Heredia, Miguel A Peña-Rico, Erick A Juarez-Arellano, David Paniagua-Vega, Eduardo Ramírez-Vargas, Saúl Sánchez-Valdes

**Affiliations:** 1 División de Estudios de Posgrado, Maestría en Ciencias Químicas, Universidad del Papaloapan, Tuxtepec Oaxaca 68301, México; 2 Centro de Investigaciones Científicas, Instituto de Química Aplicada, Universidad del Papaloapan, Tuxtepec Oaxaca, 68301, México; 3 CONACyT-UNPA, Centro de Investigaciones Científicas, Instituto de Biotecnología, Universidad del Papaloapan, Tuxtepec Oaxaca, 68301, México; 4 Centro de Investigaciones Científicas, Instituto de Biotecnología, Universidad del Papaloapan, Tuxtepec Oaxaca, 68301, México; 5 CONACyT-UANL, Departamento de Química Analítica, Facultad de Medicina, Universidad Autónoma de Nuevo León, Nuevo León, 64460, México; 6 Centro de Investigación en Química Aplicada, Saltillo Coahuila, 25294, México

**Keywords:** cancer cells, cytotoxic behavior, green synthesis, pineapple extract, silver chloride nanoparticles, silver nanoparticles, structural characterization

## Abstract

Green synthesis may be a useful approach to achieve selective cytotoxicity of silver nanoparticles on cancer cells and healthy cells. In this study, the concomitant biosynthesis of silver (Ag)/silver chloride (AgCl) nanoparticles from pineapple peel extracts and their behavior on the breast cancer cell line MCF-7 is shown. Bioreactions were monitored at different temperatures. Fourier-transform infrared spectroscopy (FTIR), ultraviolet–visible spectroscopy (UV–vis), thermogravimetric analysis (TGA), X-ray diffraction (XRD), energy-dispersive X-ray spectroscopy (EDX), and transmission electron microscopy (TEM) techniques were used to characterize nanoparticle development. The breast cancer cell line MCF-7 was used as a test model to study the cytotoxic behavior of Ag/AgCl nanoparticles and, as a counterpart, the nanoparticles were also tested on mononuclear cells. Ag/AgCl nanoparticles with spherical and triangular morphology were obtained. The size of the nanoparticles (10–70 nm) and the size distribution depended on the reaction temperature. A dose close to 20 µg/mL of Ag/AgCl nanoparticles considerably decreased the cell viability of the MCF-7 line. The best cytotoxicity effects on cancer cells were obtained with nanoparticles at 60 and 80 °C where cell viability was reduced up to 80% at a concentration of 50 µg/mL. A significant preference was observed in the cytotoxic effect of Ag/AgCl nanoparticles against cancer cells in comparison to monocytes.

## Introduction

The study of metallic nanoparticle synthesis by green methods is gaining importance, especially in cases where plant extracts are used to synthesize nanoparticles. The nanoparticles can be produced in a simple, inexpensive, and scalable way, with low reaction time and in aqueous media. Since no toxic by-products are generated, these methods are eco-friendly [1]. Silver nanoparticles (AgNPs) are commonly synthesized by green methods and, in some cases, are combined with other metals [2]. AgNPs have potential uses in biomedicine. Several authors have reported the ability of AgNPs to act as antibacterial [3–4] or as cytotoxic agents in certain cancer cell lines [5–6]. This type of application has attracted a lot of attention, given that cancer is a pathology with high incidence rates worldwide. In particular, breast cancer is highly aggressive and can metastasize, spreading to other organs through lymphatic and blood systems [7–8].

Several plant extract metabolites are known to have the ability to reduce the Ag^+^ ion of the AgNO_3_ salt to Ag^0^. In this way, silver nuclei are generated and join to form nanoparticles, which are stabilized (via capping) by the same metabolites that are involved in the oxidation–reduction process [9]. Various plant parts have been used to generate AgNPs. Amongst these parts, agricultural residues, such as fruit peels, have the potential to be used for the development of nanoparticles [10–13].

Pineapple peel has also been valued as a good source of silver salt-reducing compounds. Pineapple peel extracts have been reported to contain polyphenols such as gallic acid, catechin, epicatechin, and ferulic acid [14]. These metabolites may be potential reducing agents for the formation of AgNPs. Until now, some studies have been reported on the use of pineapple peel for the generation of AgNPs [15–18]. For example, Agnihotri et al. [15] reported photocatalytic and antibacterial abilities of AgNPs synthesized from pineapple peel. They demonstrated the formation of spherical AgNPs with an average size of 14–20 nm by monitoring the pH values of the reaction and the concentration ratio between the precursor and the extract. Baran et al. [16] investigated the antibacterial and anticancer properties of AgNPs synthesized from pineapple peel. The authors reported a favorable antimicrobial activity at low concentrations of AgNPs. Das et al. [17] found that AgNPs synthesized in the same way have high antidiabetic potential and high cytotoxicity against HepG_2_ cancer cells in a dose-dependent manner.

Based on the aforementioned findings, and considering the high content of phenolic compounds in the pineapple peel, which function as reducing agents of silver salt, the present study shows biosynthesis of Ag/AgCl nanoparticles using a pineapple peel extract. The study was conducted by monitoring biosynthesis temperature, considering that this variable has an important influence on the formation of nanoparticles. To verify the biological behavior of the obtained Ag/AgCl nanoparticles, their cytotoxic activity in the MCF-7 breast cancer cell line was investigated.

The novelty of this work is based on three major points. Firstly, by taking advantage of using pineapple waste, green synthesis methods were applied to obtain silver nanoparticles. In this way, an alternative use of agricultural residues was created, providing added value to fruit products. The second point is the obtaining of metallic Ag nanoparticles combined with AgCl, where AgNPs were formed by reducing compounds of the extract. Thus, the formation of AgCl was due to the availability of chlorine salts in pineapple peels. The third novelty shown in this work is that the cytotoxic activity of Ag/AgCl nanoparticles on breast cancer cells is dependent on the biosynthesis temperature. Consequently, its effect is different in cancer cells in comparison to healthy cells (monocytes). This result may give rise to a new system with cytotoxic selectivity. The goal of this study is to contribute to the generation of alternative materials for therapeutic applications, especially those that mitigate diseases.

## Results and Discussion

### Ag/AgCl biosynthesis

It has been reported in the literature that pineapple contains several phenolic compounds [14,19], which could act as reducing agents of silver salt. For this reason, the amount of phenolic compounds in the pineapple peel extract was quantified. The total phenolic content (TPC) in the pineapple peel extract was 24.66 ± 1.03 mg Catechin/g Ext, and the total flavonoid content (TFC) was 0.62 ± 0.21 mg Rutin/g Ext. The extract has a higher phenolic content in comparison to its flavonoid content. Li et al. [14] reported that some phenolic compounds, such as gallic acid, catechin, epicatechin, and ferulic acid are present in pineapple peel extracts. On the other hand, Steingass et al. [19] reported an extensive phytochemical study, by HPLC-DAD-ESI-MS*^n^* and GM-MS, of pineapple phenolic compounds including those in pineapple peel.

**Figure 1:**
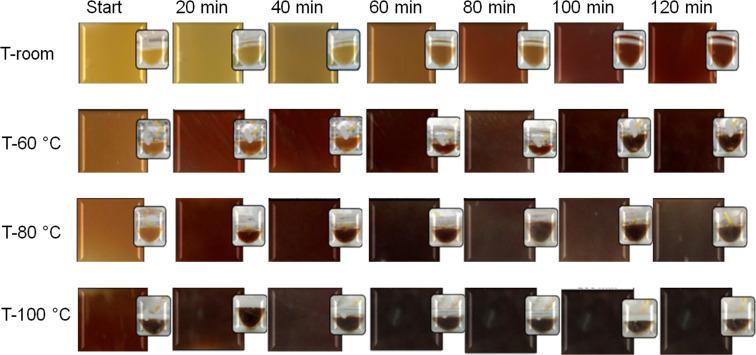
Photographs of Ag/AgCl nanoparticle biosynthesis from pineapple peel extracts. The photographs were taken at specified times and temperatures.

Photographs of Ag/AgCl biosynthesis using pineapple peel are shown in Figure 1. Photographs were taken every 20 min up to 120 min for each reaction. The reaction temperatures are expressed as room temperature, 60, 80, and 100 °C, respectively. It can be clearly observed that as the reaction proceeds, a color change from yellow to reddish brown is produced, similar to that reported in the literature [20]. This behavior is the first evidence that the reaction between the biowaste and the silver salt is taking place. In a previous report [21] these color changes were also observed during the formation of AgNPs from the *Stevia rebaudiana* extract. This behavior was attributed to the gradual formation of Ag nanoparticles, and the morphological changes that occur during biosynthesis. It is important to note that temperature has a considerable effect on biosynthesis. For example, based on its coloration, the reaction at room temperature after 120 min has a similar result as a reaction at a temperature of 60 °C or higher after only 20 min.

According to the literature, AgNPs synthesized from plant extracts can be directly produced at room temperature [22–24]. In this way, the ability of secondary metabolites of plant extracts to reduce precursor metal salts to particles with zero charge, and at the same time stabilize nanoparticles already formed, has been demonstrated. Despite this, the phenomenon of interaction of the chemical species of the extracts with the precursor salt could be enhanced depending on the temperature, since the kinetic and thermodynamic effects in the reaction system could be maximized [25]. Consequently, the formation of nanoparticles could be faster or more efficient in terms of size and shape of the nanoparticles.

### Crystalline behavior

**Figure 2:**
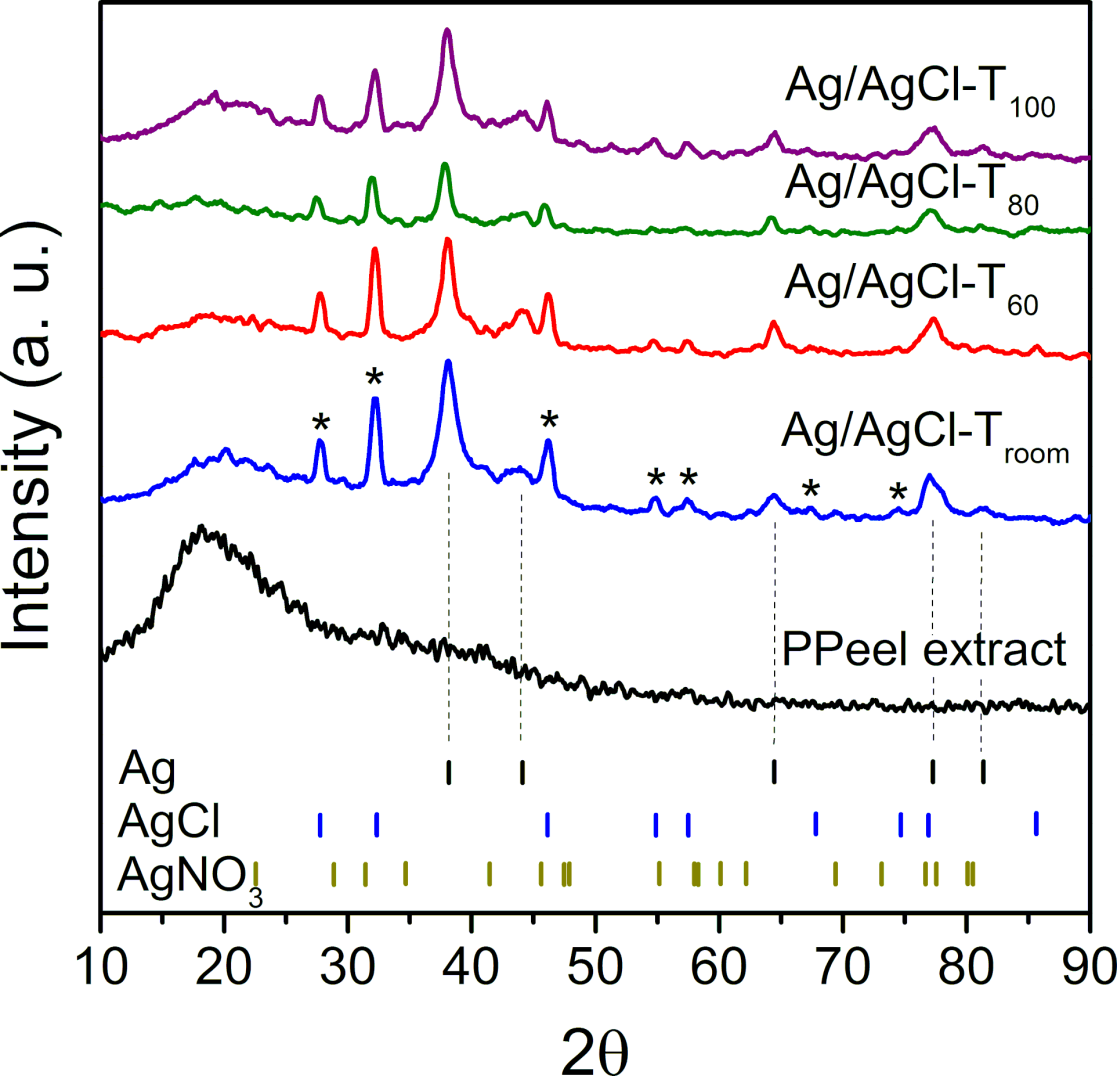
Diffraction patterns of the reaction products Ag/AgCl-T_room_, Ag/AgCl-T_60_, Ag/AgCl-T_80_, and Ag/AgCl-T_100_. Metallic Ag, AgCl, and AgNO_3_ signals are set as references. The peaks marked with asterisks are related to the crystallographic planes of AgCl. The dashed lines represent the crystallographic signals of metallic Ag.

In all of the reactions, the X-ray diffraction patterns shown in Figure 2 confirm the transformation of AgNO_3_ into metallic Ag. The characteristic peaks of AgNO_3_ salt and metallic Ag are indicated by short lines and can be used as a reference for comparison with the diffraction peaks obtained from the reaction products. In addition to the Ag reference pattern, an AgCl reference pattern is also attached. The latter was added because the experimental diffractograms of the reaction products showed characteristic peaks for Ag and AgCl. The Ag reference pattern was obtained from card number 00-04-0783 (Joint Committee on Powder Diffraction Standards, JCPDS). This pattern represents the peaks corresponding to the crystallographic planes (111), (200), (220), and (331) of the Cu-type face-centered cubic crystal structure of metallic Ag. The pattern for AgCl was taken from letter number 56540 (Inorganic Crystal Structure Database, ICSD). This pattern corresponds to the crystallographic planes (111), (200), (220), (311), and (222) of the NaCl-type face-centered cubic crystal structure.

According to the results, the pineapple peel extract (PPeel extract) is amorphous (i.e., does not show any signal of molecular order). The lack of AgNO_3_ salt in the reaction products can also be observed. Hence, the diffraction patterns of the products obtained, regardless of the temperature used, show a combination of well-defined peaks of metallic Ag indicated with dashed lines and AgCl indicated with asterisks.

In an earlier work, the same combination of Ag and AgCl signals was obtained using extracts of *Stevia rebaudiana* [21]. In that report, the effect of the ratio between metallic AgNPs and AgClNPs on the morphology and dispersion in a thermoplastic starch matrix was demonstrated. At the same time, the nanodispersion behavior was related to the cytotoxic activity on cancer cells. The best results were obtained with the combination of nanoparticles mainly with AgCl. In other studies, the formation of Ag and AgCl signals has also been detected by XRD when using plant extracts [26–28].

As mentioned by Raven [29], Cl^−^ is an essential micronutrient for oxygenic photosynthetic organisms and is found in the environment in concentrations higher than those required by plants. Teixeira et al. [30] reported that the use of potassium chloride as a source of potassium for pineapple crops increases the availability of Cl^−^ in the soil and in the leaves of the plant. In this case, it is proposed that the synthesis of AgCl occurred by the interaction of the chloride ions present in the pineapple peel with the silver ions of AgNO_3_ as other authors have pointed out [31–32]. The high concentration of chloride ions in pineapple peel may be due to the use of fertilizers with a high chloride content during pineapple cultivation. It is also considered that by adding silver salt to the extract, competitive reactions occur for the formation and stabilization of Ag or AgCl nanoparticles, giving rise to a concomitant generation of both silver species.

The crystallite size of Ag and AgCl was calculated from the XRD data and using the Scherrer equation, *D* = (*K*λ)/(βcosθ), where *D* is the average crystallite size, *K* is the shape factor (a value of 0.94 was used for this analysis), λ is the wavelength of the X-ray radiation (which is 0.15418 nm), β is the full width at half maximum (FWHM) in radians, and θ is the Bragg angle. In addition, using the Match!^®^ Software, the content of Ag and AgCl was also calculated, and the results are shown in Table 1.

**Table 1:** Content and crystallite size of Ag and AgCl calculated from XRD results.

Product	Crystallite size (nm)		Content (%)	
	Ag	AgCl	Ag	AgCl

Ag/AgCl-T_room_	6.01	11.54	54.3	45.7
Ag/AgCl-T_60_	7.32	11.70	46.3	53.7
Ag/AgCl-T_80_	7.87	11.46	43.1	56.6
Ag/AgCl-T_100_	6.32	10.67	52.1	47.9

According to Table 1, the change in crystallite size as a function of temperature is more noticeable for Ag than for AgCl, where an increasing trend in Ag crystallite size is observed up to 80 °C. It is speculated that at this temperature, the separation of salt ions and the interaction with reducing biological compounds are favorable, which enables the formation of a Ag nuclei and the growth of crystallites. In contrast, higher temperatures result in a higher reaction rate, causing a rapid conversion of Ag^+^ into metallic Ag [33], as shown by the color change in Figure 1. This latter may be the cause of the impediment in the growth of crystallites at 100 °C. On the other hand, the different contents of Ag and AgCl depend on the presence of chloride ions in the extract and their ability to form AgCl instead of metallic Ag.

**Figure 3:**
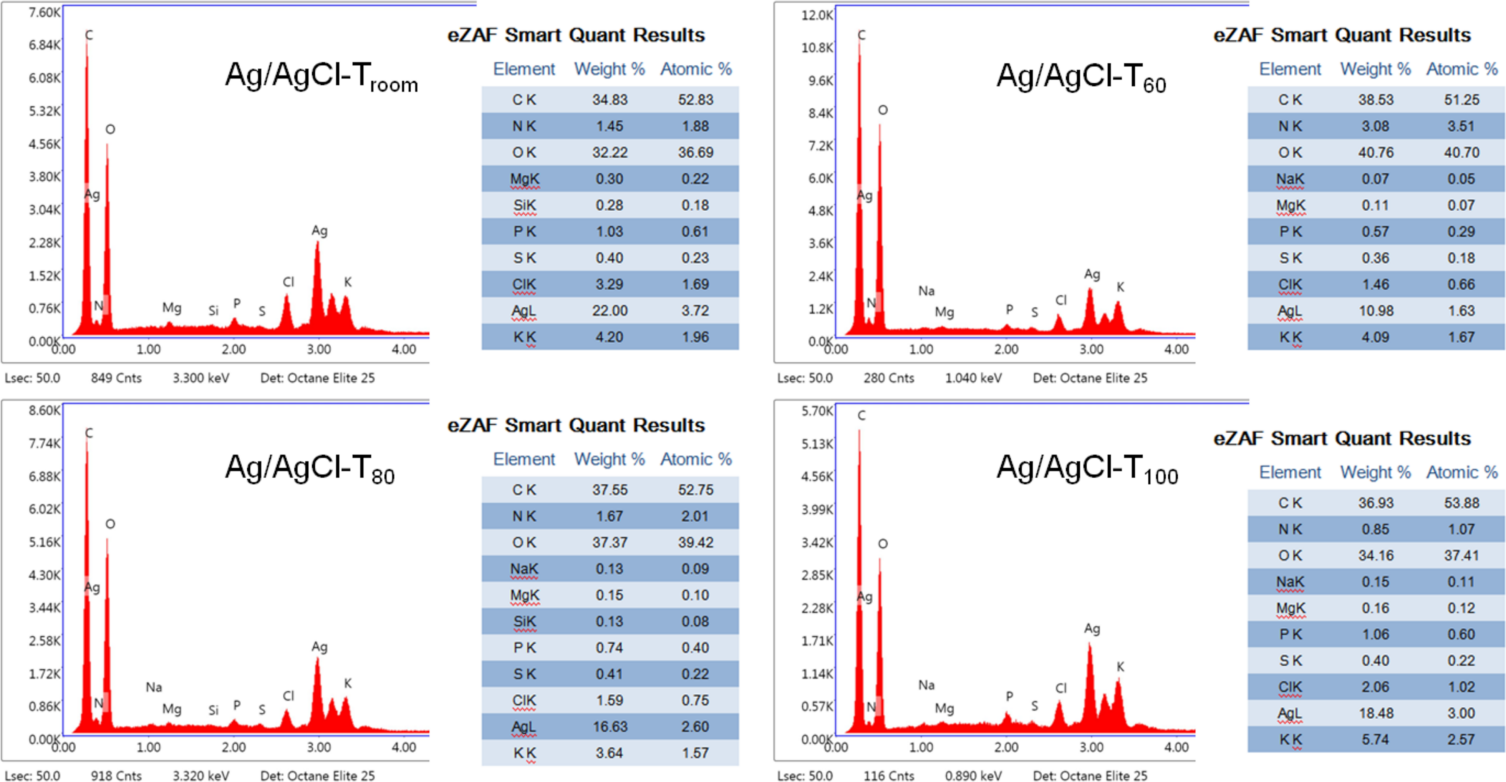
EDX analysis and quantification of Ag/AgCl products at different synthesis temperatures.

The qualitative and quantitative energy-dispersive X-ray (EDX) chemical analysis of Ag/AgCl products at different temperatures is shown in Figure 3. Consistently, the presence of Ag and Cl is evident. Likewise, characteristic elements of the compounds present in the pineapple peel extract are revealed, which act as capping agents for the nanoparticles. Silver is one of the most concentrated elements since it exists as metallic Ag and as an ion in AgCl. According to the quantitative results, Ag/AgCl-T_room_ and Ag/AgCl-T_100_ have the highest Ag content. In contrast, Ag/AgCl-T_60_ and Ag/AgCl-T_80_ have the lowest Ag concentrations. This result is consistent with the data determined from the XRD diffractograms shown in Table 1. Therefore, as mentioned before, the temperature affects the generation of AgNPs. However, for this particular case, the temperature also contributes to the formation of AgCl nanoparticles.

### Spectroscopic characterization

**Figure 4:**
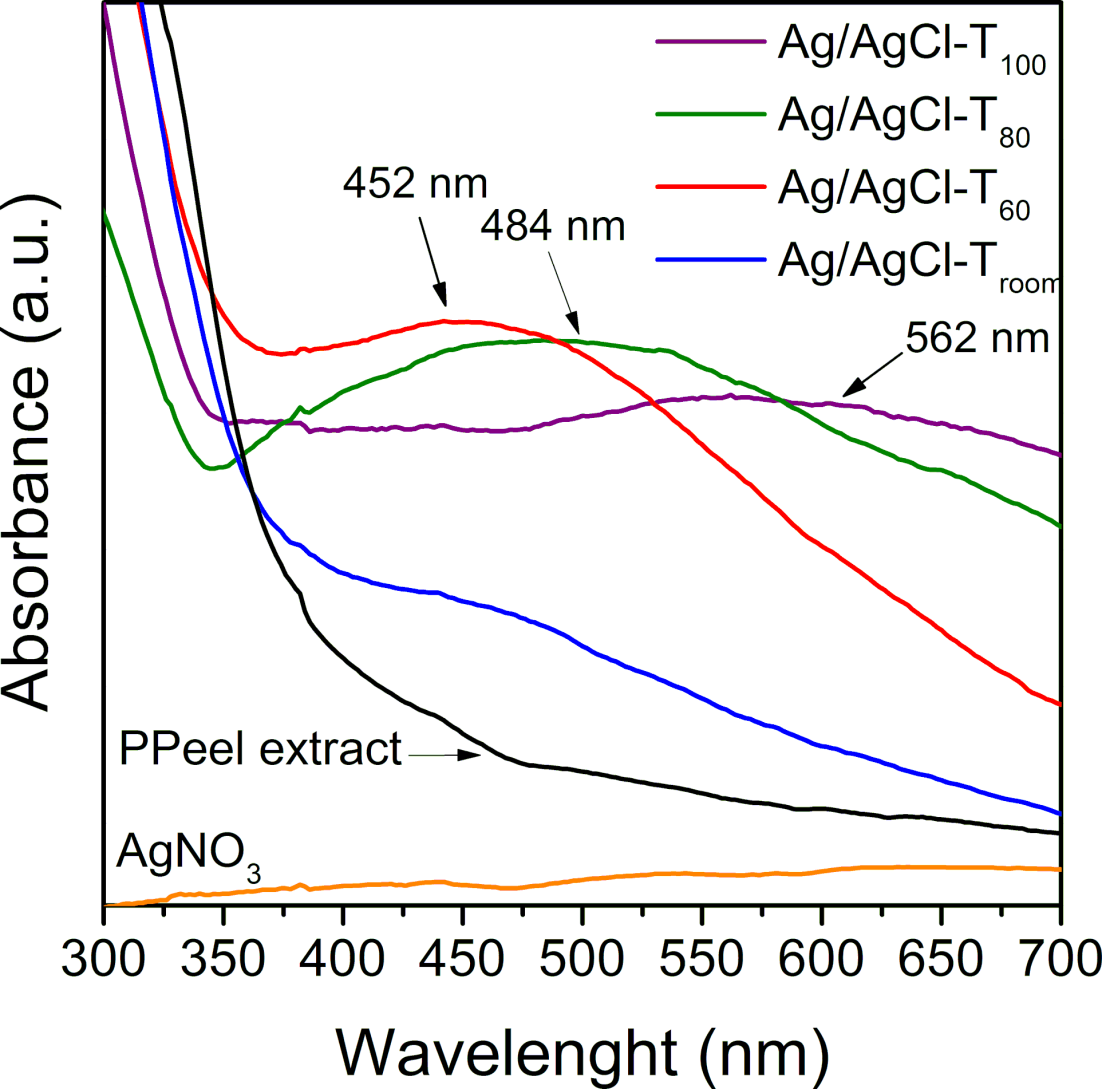
UV–visible spectroscopy of Ag/AgCl nanoparticle biosynthesis. The maximum absorption peaks are denoted by the arrows.

It is known that the interaction of light with free electrons in an Ag nanoparticle can give rise to a collective oscillation known as surface plasmon effect [34]. This effect can be monitored by UV–vis spectroscopy, where metal nanoparticles absorb radiation at different wavelengths depending on their size [36]. The UV–vis absorption spectra of the reactions at different temperatures are shown in Figure 4. The absence of absorption of visible radiation is evident in the AgNO_3_ salt and in the pineapple peel extract. Instead, reactions at different temperatures showed absorption in this region of the electromagnetic spectrum. Furthermore, differences in the maximum values of the absorption curves can be observed. Although in the reaction at room temperature (Ag/AgCl-T_room_) the absorption maximum was not obvious, and it was in the case of the other reactions, the absorption maxima were easily identified. Ag/AgCl-T_60_ shows absorption maximum at 452 nm, Ag/AgCl-T_80_ at 484 nm, and Ag/AgCl-T_100_ at 562 nm. For AgNPs, the maximum absorption signal is typically in the range of 400–500 nm [31]. Absorption trends at longer wavelengths are associated with increasing particle size [35–36]. It is also evident from Figure 4 that the absorption peak for the reaction at 100 °C (Ag/AgCl-T_100_) is broader than that of the reactions at 60 and 80 °C. This behavior may be related to a larger size distribution of the nanoparticles. Hence, the uniformity of the nanoparticles is higher at 60 and 80 °C. This result is consistent with Nayak et al. [37], who reported an optimal temperature of 80 °C for Ag nanoparticle formation using extracts from *Cucurbita maxima*, *Moringa oleifera,* and *Acorus calamus*.

**Figure 5:**
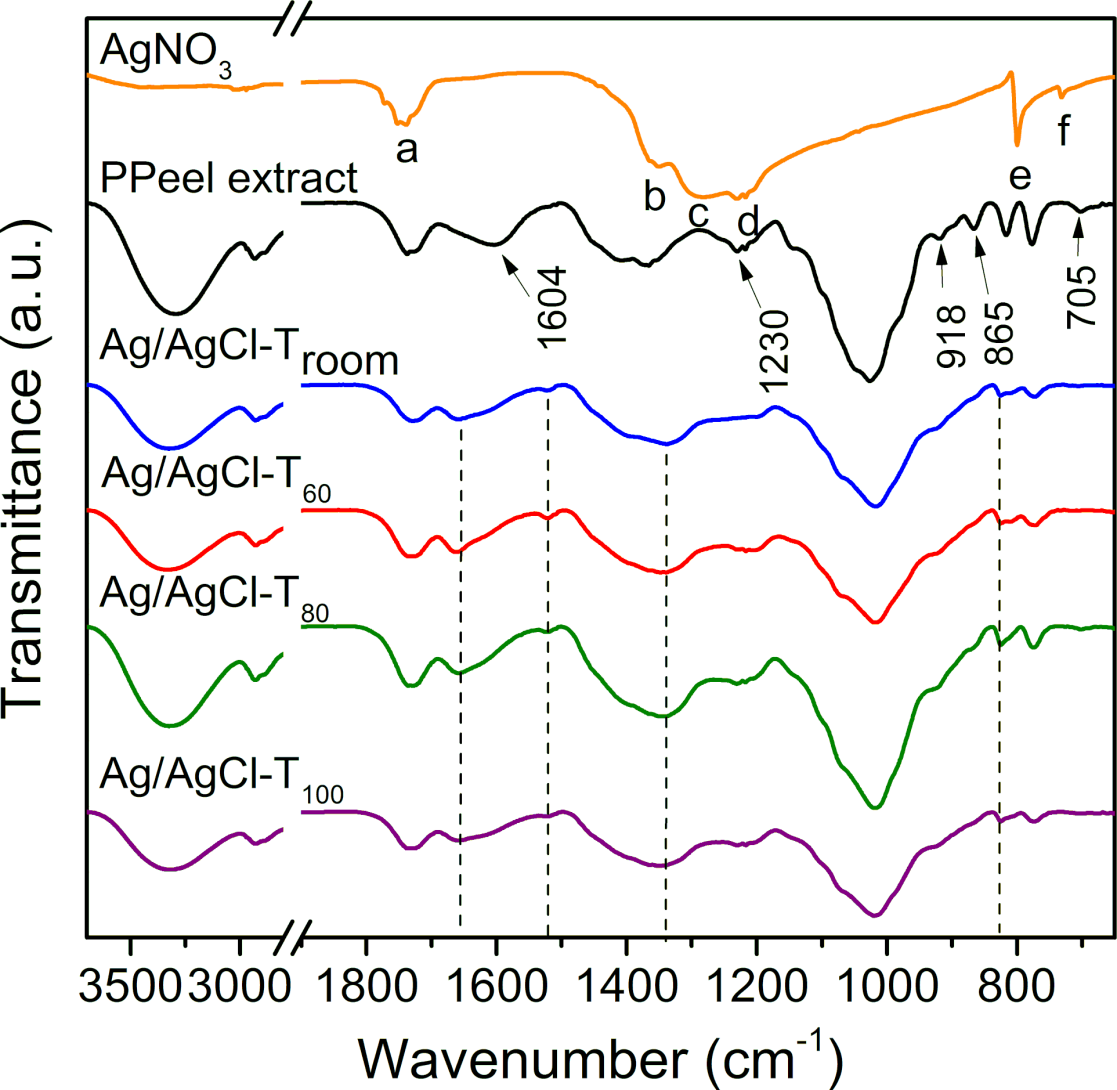
FTIR spectra of Ag/AgCl nanoparticles. The functional groups of the AgNO_3_ salt are represented by the letters a–f. The wavenumber values of the functional groups of the pineapple peel extract which disappear in the reactions are denoted by arrows. New absorption peaks in the reaction products are shown by dashed lines.

The FTIR results are shown in Figure 5. The spectra of the precursor salt and pineapple peel extract are shown for signal comparison. In the spectrum of the AgNO_3_ salt, some signals were observed at (a) 1746 cm^−1^ corresponding to the symmetric tension and deformation in the N–O plane; (b) 1360 cm^−1^, (c) 1284 cm^−1^, and (d) 1230 cm^−1^ related to the asymmetric tension of N–O; (e) 800 and (f) 732 cm^−1^ related to the deformation and oscillation in the N–O plane. The following bands are found in the pineapple peel extract spectrum: 3300 cm^−1^ corresponds to O–H stretching in phenolic compounds; 2930 cm^−1^ is related to C–H stretching in any of the metabolites; 1737 cm^−1^ corresponds to C=O stretching in tannins; 1604 cm^−1^ and 1410 cm^−1^ are related to C=C stretching of aromatic rings in flavonoids, terpenes, tannins, and gallic acid; 1230 cm^−1^ corresponds to the tension of tertiary alcohols and flavonoids; 1026 cm^−1^ is related to C–O vibration in tannins and flavonoids; and 918 cm^−1^, 865 cm^−1^, and 705 cm^−1^ correspond to out-of-plane C–H vibration in gallic acid and catechin. According to the spectra shown in Figure 5, the formation of Ag/AgCl is evidenced by three phenomena. First, the AgNO_3_ salt reacted completely as the characteristic absorption signals denoted by letters a–f in the AgNO_3_ spectrum did not appear in the reaction products. Second, some of the absorption bands (indicated by arrows) of the pineapple peel extract disappeared. Third, new absorption bands are generated at 1663 cm^−1^, 1522 cm^−1^, 1340 cm^−1^, and 825 cm^−1^ and are indicated by dashed lines in the spectra. This behavior occurs regardless of the reaction temperature used. According to the literature, pineapple peel contains several chemical species, especially phenolic and polyphenolic substances such as catechin, epicatechin, gallic acid, and ferulic acid [14]. These metabolites have reducing capacity, so it is hypothesized that this allows for the reduction of Ag^+^ to Ag^0^. The changes observed in the absorption bands of the reaction products are also a consequence of the capacity of the extract metabolites to act as capping agents for the formation of nanoparticles.

### Morphological characterization

**Figure 6:**
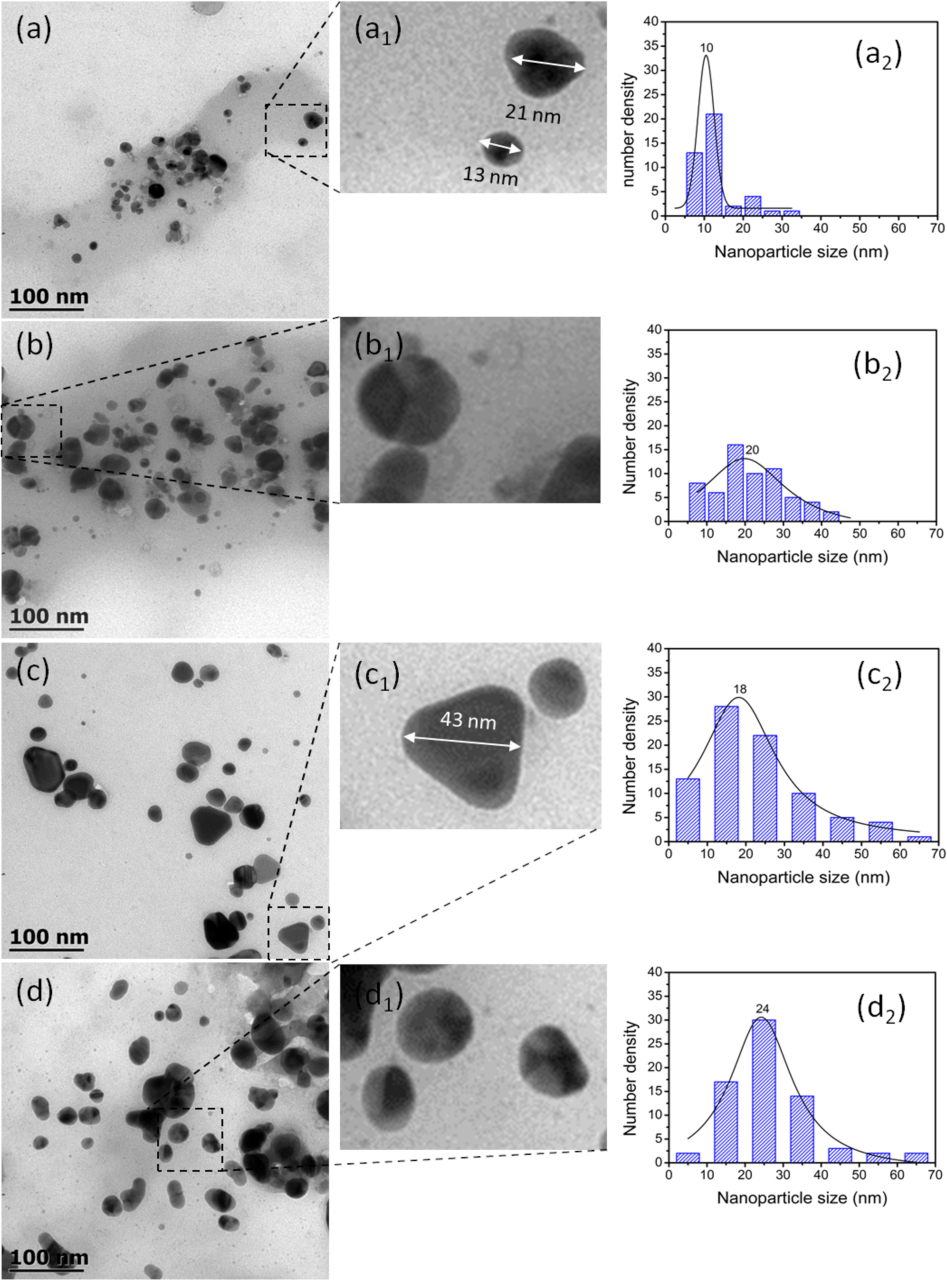
TEM micrographs of nanoparticles obtained at different temperatures. (a) Ag/AgCl-T_room_, (b) Ag/AgCl-T_60_, (c) Ag/AgCl-T_80_, and (d) Ag/AgCl-T_100_. Micrographs marked with subscript 1 are magnifications of some nanoparticles. In (a_1_) and (c_1_), sizes are indicated by double-headed arrows. The graphs with subscript 2 represent nanoparticle size distribution.

Although the data obtained by XRD and FTIR do not show significant differences in the formation of nanoparticles as a function of temperature, the micrographs obtained by TEM in Figure 6 show different behaviors both in size and shape of the nanoparticles with respect to temperature. These results are consistent with UV–vis spectra shown in Figure 4, where each curve has a different maximum depending on the reaction temperature. If the size of the nanoparticles is considered, as the reaction temperature increases, the nanoparticles become larger or the size distribution becomes broader. At room temperature and at 60 °C, the nanoparticles are less than 50 nm in size, but at 80 °C the nanoparticles reach a size of 70 nm. The shape of the nanoparticles is also modified, going from mostly spherical particles to a better defined morphology, as seen at 80 °C (Ag/AgCl-T_80_). It is evident that at a temperature of 80 °C, in addition to spherical particles, triangular plate-like nanoparticles with well-defined and equal sides are obtained (see Figure 6C_1_). This result is consistent with Hyllested et al. [38] who also obtained AgNPs with triangular morphology using pineapple extract. The synthesis temperature plays an important role in the formation, growth, and size distribution of nanoparticles, as mentioned by Jiang et al. [39]. They reported the coexistence of triangular and spherical silver particles of different sizes obtained at temperatures ranging from 17 to 55 °C. According to the morphological results shown here, as the temperature increases the reaction rate also increases, favoring interactions between the reducing biocompounds and the precursor salt. Concomitantly, the particle size increases, as shown by the trend of images a_2_–d_2_ in Figure 6. However, the particle size distribution also increases because the higher the reaction rate, the greater the formation of silver crystals of different sizes. In the micrographs in Figure 6, it can be seen that the nanoparticles are embedded in a disordered system, with very low electron density. This system could be made of organic molecules that act as capping materials for nanoparticles, as described in the literature [24,40].

### Thermal behavior

**Figure 7:**
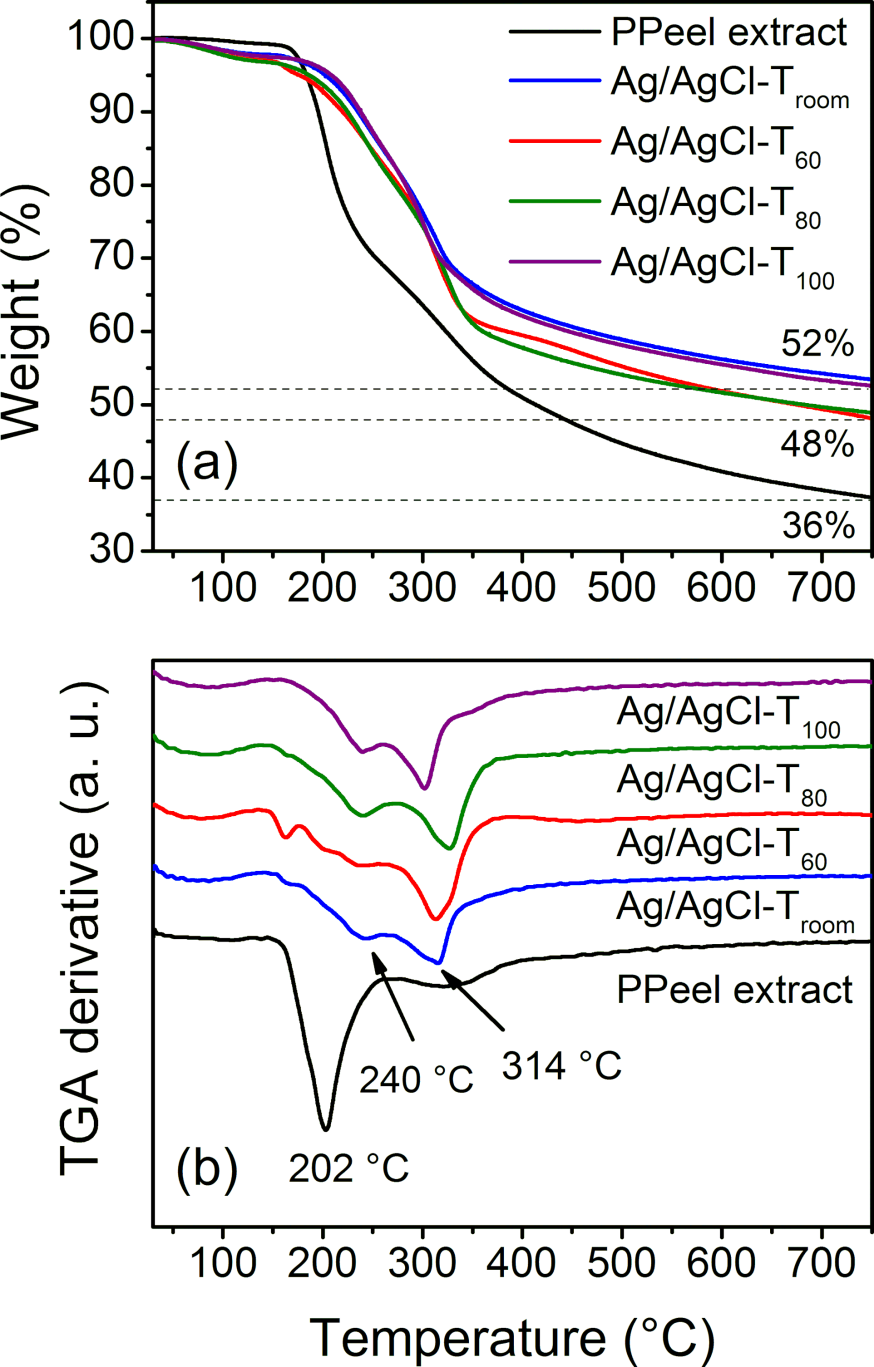
(a) TGA data and (b) weight loss derivatives of Ag/AgCl-T_room_, Ag/AgCl-T_60_, Ag/AgCl-T_80_, and Ag/AgCl-T_100_. The percentage of residual material after 700 °C is shown in (a). The average degradation temperature signals are indicated by the arrows in (b).

The weight loss curves and derivatives calculated from the thermogravimetric analysis data of the reaction products are shown in Figure 7a and Figure 7b, respectively. In the thermogram of the pineapple peel extract, denoted as PPeel extract, two stages of degradation of the metabolites were observed. The first degradation signal is very well defined and occurs at 202 °C, with an approximate mass loss of 35%. The second stage of degradation occurs in a temperature range of 277–395 °C, with an average temperature of 330 °C and an approximate mass loss of 15%. As the temperature increased, the sample continued to degrade such that at 750 °C, 36% of char residue remained (Table 2).

**Table 2:** Thermal degradation data of pineapple peel extract and reaction products.

Sample	First weight loss			Second weight loss			Residual content (%)
	*T*_deg_ (°C)	Temperature range	Weight loss (%)	*T*_deg_ (°C)	Temperature range	Weight loss (%)	

PPeel extract	202	160–262	35	330	277–395	15	36
Ag/AgCl-T_room_	240	175–269	20	314	274–339	13	52
Ag/AgCl-T_60_	240	175–269	20	314	274–363	20	48
Ag/AgCl-T_80_	240	175–269	20	326	274–368	21	48
Ag/AgCl-T_100_	240	175–269	20	301	274–339	13	52

In the thermograms of the reaction products, two stages of degradation are also shown, except for Ag/AgCl-T_60_, where a small additional peak appears at 163 °C, possibly from volatile organic molecules. The first degradation in the reaction products is at 240 °C. The second degradation peak for both Ag/AgCl-T_room_ and Ag/AgCl-T_60_ is found at 314 °C. In the case of the Ag/AgCl-T_80_ reaction product, the degradation signal is at 326 °C and in Ag/AgCl-T_100_ the degradation temperature is 301 °C (Table 2).

Based on the results in Figure 7, it is remarkable that the reaction products have a higher thermal stability than that of the pineapple peel extract. This behavior can be attributed to two complementary situations. The first is a change in the chemical structure of the organic compounds used in the formation and capping of Ag/AgCl nanoparticles as observed by FTIR spectroscopy (Figure 5). The reducing species are oxidized and as a consequence have greater thermal stability. Hence, the higher temperature-shifted TGA curves show the thermal behavior of the modified metabolites during chemical reaction. The results are also consistent with Reddy et al. [41], who reported that phytochemicals capped on nanoparticles support thermal stability upon temperature changes. The second situation is the thermal barrier that Ag/AgCl nanoparticles themselves, based on their intrinsic characteristics, provide to the system. This last proposal is consistent with Rhim et al. [42], who stated that the increased thermal stability of the agar/AgNPs composite films is due to metallic silver being more thermally stable. On the other hand, throughout the thermal test, no evidence of decomposition of the AgNO_3_ precursor salt (decomposition temperature is approx. 500 °C) is observed [43]. Hence, and as indicated by the FTIR and UV–vis results, the AgNO_3_ salt was converted into Ag/AgCl nanoparticles. Furthermore, Figure 7 shows that the residual content in the reaction products is higher than that in the extract, illustrating the maximum percentage of biosynthesized Ag/AgCl nanoparticles (52% − 36% = 16%).

The degradation of the product with respect to temperature exhibited a behavior similar to that of nanoparticle biosynthesis, as shown by XRD, EDX, and TEM. That is, there is a difference in the degradation at intermediate temperatures (Ag/AgCl-T_60_ and Ag/AgCl-T_80_) with respect to the products synthesized at room temperature and at 100 °C (Ag/AgCl-T_room_ and Ag/AgCl-T_100_). This behavior particularly occurs in the second degradation and in the residual content. Therefore, this trend is probably related to the changes after the biological compounds act as reducing agents, as well as to changes in the shape, size, and size distribution of the resulting Ag nanoparticles.

### Cytotoxic behavior

**Figure 8:**
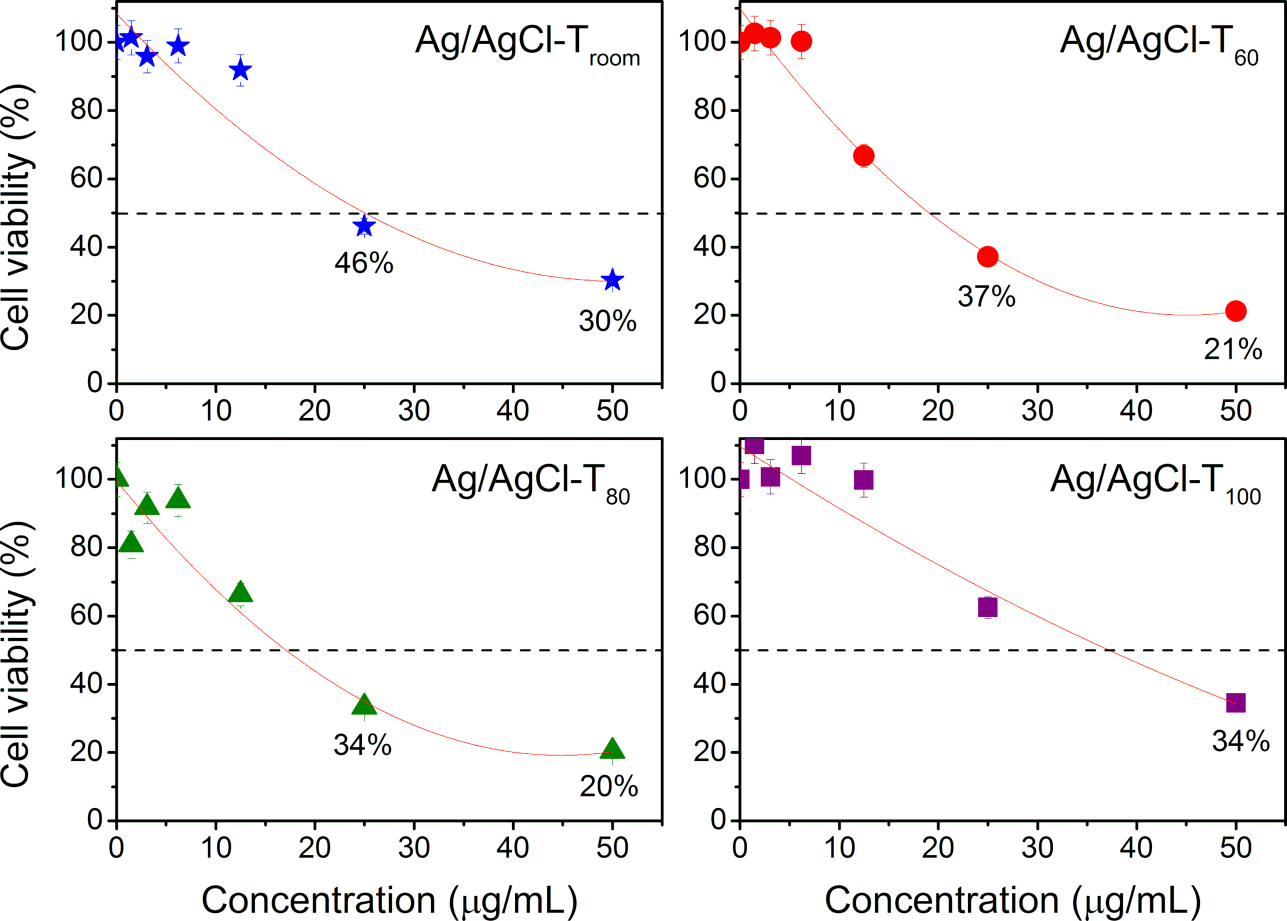
Cell viability assay of MCF-7 cells with Ag/AgCl nanoparticles obtained at room temperature, 60, 80, and 100 °C. The horizontal dashed line indicates 50% of cell viability. In all systems, cell viability at nanoparticle concentrations of 25 and 50 µg/mL is indicated, except for Ag/AgCl-T_100_, since only cell viability at the concentration of 50 µg/mL is below 50%.

It has been reported that several cytotoxic mechanisms of AgNPs can cause DNA, mitochondrial, and cell membrane damage as well as apoptosis [44]. Here, the cytotoxicity results of Ag/AgCl nanoparticles on MCF-7 breast cancer cells are shown in Figure 8. For each system of nanoparticles produced at different temperatures, cell viability is related to nanoparticle concentration. In all cases, cell viability decreased in a dose-dependent manner (i.e., cell death was progressive with increasing concentration). Cell viability was below 50% for Ag/AgCl-T_room_, Ag/AgCl-T_60_, and Ag/AgCl-T_80_ systems at concentrations below 25 µg/mL. Reactions at room temperature, 60, 80, and 100 °C achieved IC_50_ values of 24, 19, 17, and 36 µg/mL, respectively. Hence, the best behaviors occur with the nanoparticles formed at 60 and 80 °C. The results were favorable for all systems tested at 50 µg/mL. Here, the Ag/AgCl-T_60_ and Ag/AgCl-T_80_ systems also showed the best cytotoxic behavior, with cell viability of 21 and 20%, respectively. This response means that between 60 and 80 °C, it is beneficial to generate nanoparticles with a size and morphology suitable to induce cancer cell cytotoxicity.

According to the results obtained by TEM (Figure 6), at 60 °C spherical nanoparticles with a size range between 10 and 40 nm (size average of 20 nm) were obtained. At 80 °C, spherical nanoparticles and triangular plate-like nanoparticles were formed with a wider size range of 5–60 nm (size average of 18 nm). Even so, the size of the triangular plates is larger than 40 nm. Therefore, the morphology and size of nanoparticles are thought to have a great influence on the cytotoxicity of cancer cells. This result is consistent with Park et al. [45], who reported that the size of AgNPs is an important factor in cytotoxicity, inflammation, and genotoxicity. In this sense, AgNPs have been shown to induce cytotoxicity through apoptosis and necrosis in different cell lines [46].

**Figure 9:**
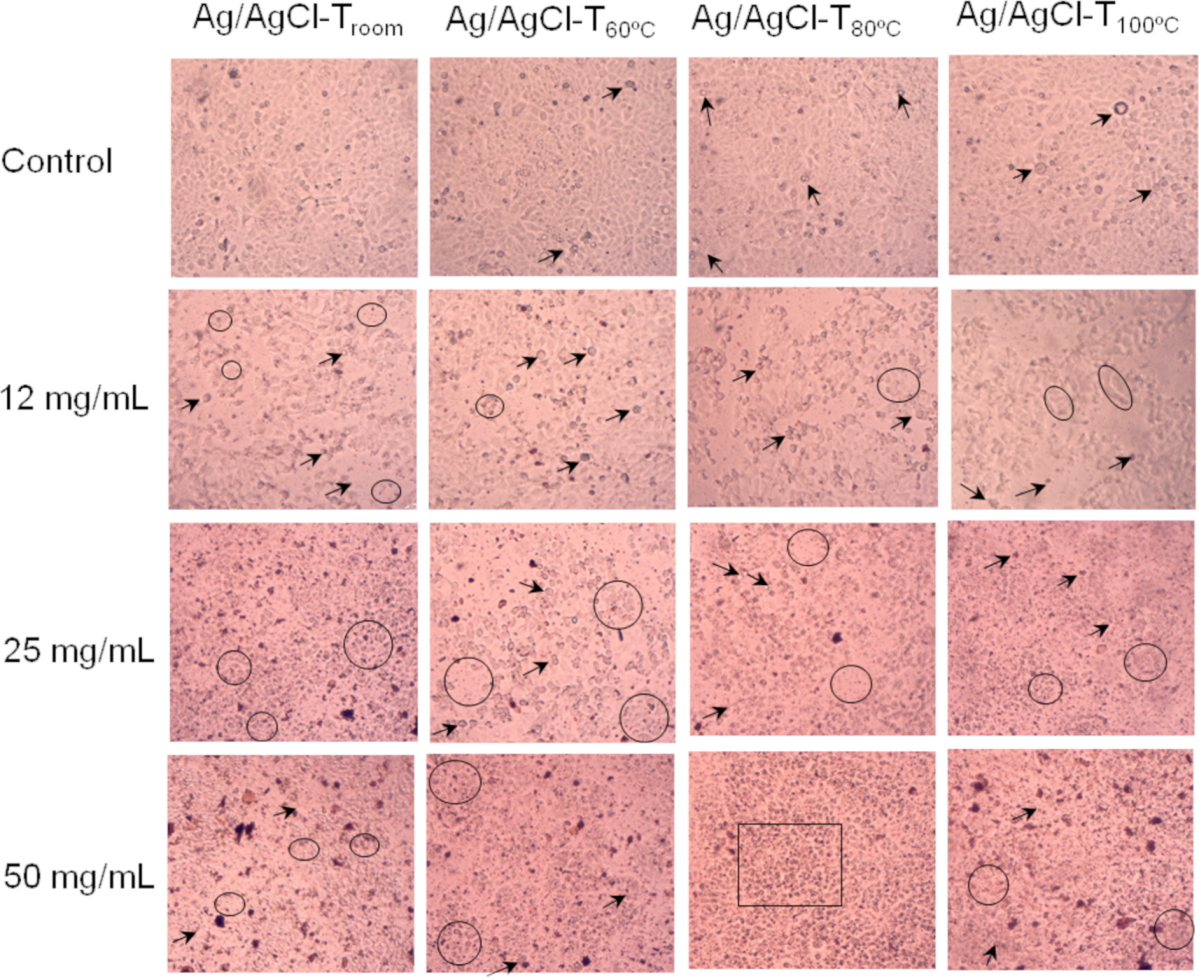
Microscopic observation of control MCF-7 cells and MCF-7 cells with Ag/AgCl nanoparticles after 24 hours of exposure. The MCF-7 cell line shows increased cell area or morphological changes (black circles) and formation of apoptotic bodies (arrows). At high concentrations, a large number of cells in a necrotic state were detected (black box).

Microscopic analysis (Figure 9) clearly shows that the morphological changes depend on the synthesis temperature and dosage of Ag/AgCl nanoparticles. The data show that the formation of apoptotic bodies (arrows) and the growth of cellular regions (black circles) are integral and characteristic of apoptosis. At high concentrations, however, necrosis (black box) predominates, where the formation of cellular debris and damage to the cell membrane are detected. Depending on the level of stress exerted on the cell, this behavior will trigger cell death [47]. Çìftçì et al. [48] suggested that AgNPs induce apoptosis and necrosis in MCF-7 cells at lower concentrations, but induce necrosis only at higher concentrations.

**Figure 10:**
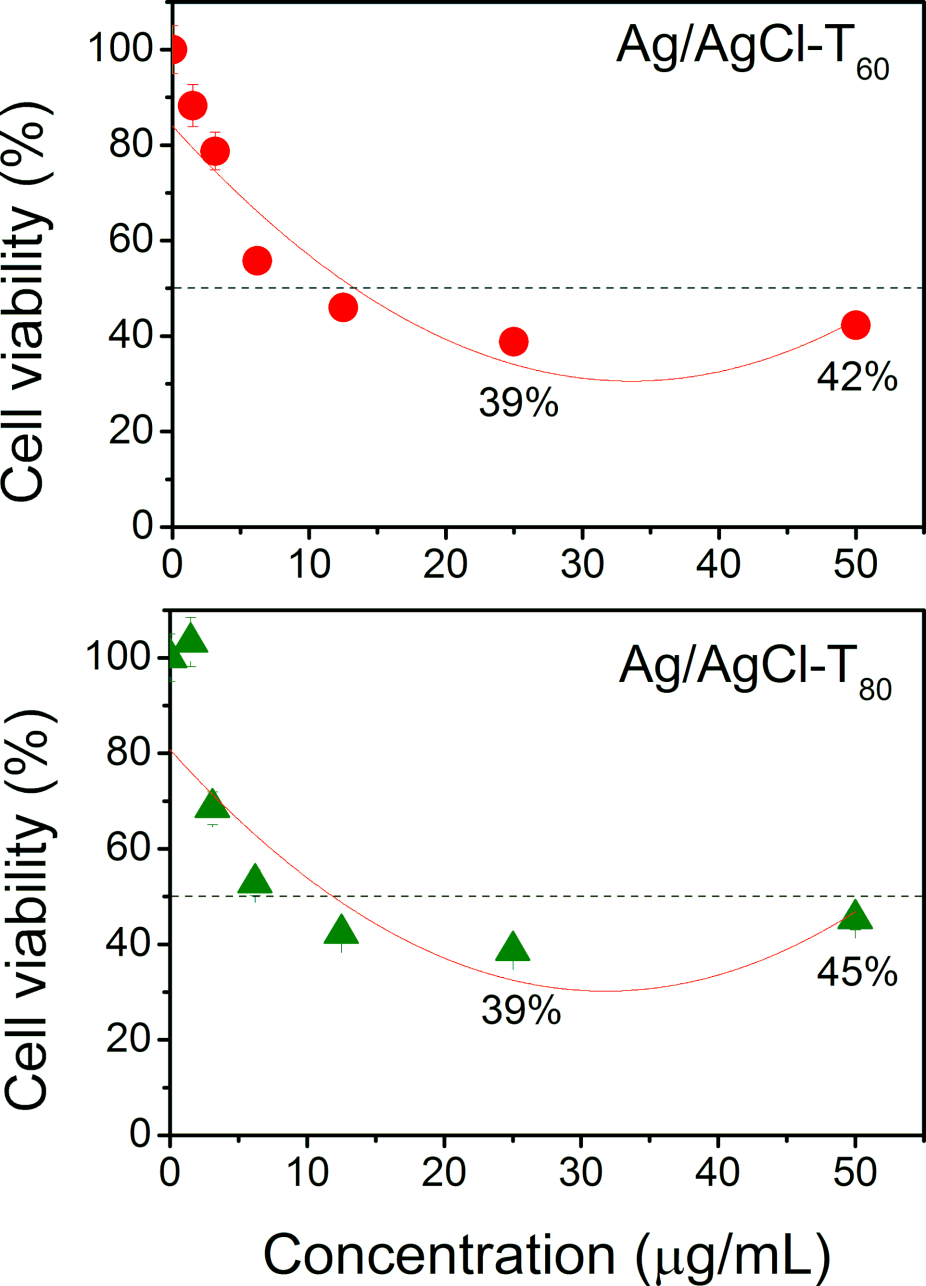
Cell viability tests on mononuclear cells (monocytes) with Ag/AgCl nanoparticles obtained at 60 and 80 °C. Cell viability at 50% is marked with a horizontal dashed line. Cell viability at 25 and 50 µg/mL is indicated.

Despite the strong cytotoxic activity of nanoparticles on MCF-7 cells, it was necessary to examine whether there is any effect on healthy cells. The systems with lower IC_50_ (Ag/AgCl-T_60_ and Ag/AgCl-T_80_) were tested in mononuclear cells, particularly in monocytes. The results of the cytotoxic behavior of these systems are shown in Figure 10. It is evident that nanoparticles obtained at temperatures of 60 and 80 °C were also cytotoxic to monocytes at concentrations of 25 µg/mL. In fact, their IC_50_ was lower than that of MCF-7 cells at 13 and 12 µg/mL, respectively. Interestingly, an unexpected result was that for concentrations above 35 µg/mL, especially at 50 µg/mL, the cytotoxic effect of nanoparticles was more pronounced on cancer cells than on monocytes. The difference was 21% for AgNPs-T_60_ and 25% for AgNPs-T_80_. In other words, at concentrations close to 50 µg/mL, the cytotoxic action of the nanoparticles becomes selective. This result is possibly due to the fact that a higher content of nanoparticles prevents cell proliferation in neoplastic cells. MCF-7 cells are known to overexpress matrix metalloproteinases (MMPs), the activity of which is favored by reactive species, and they have been shown to be directly involved in death mechanisms such as apoptosis, causing damage at the cell membrane level. In contrast, in monocytes, which are also high in MMPs, their activation mechanism is largely dependent on the production of NOs. Perhaps this fact is an explanation for the selectivity of the findings. However, a more in-depth study of this mechanism is necessary. These data have never been reported before, so their mechanistic interpretation and understanding requires further investigation.

**Figure 11:**
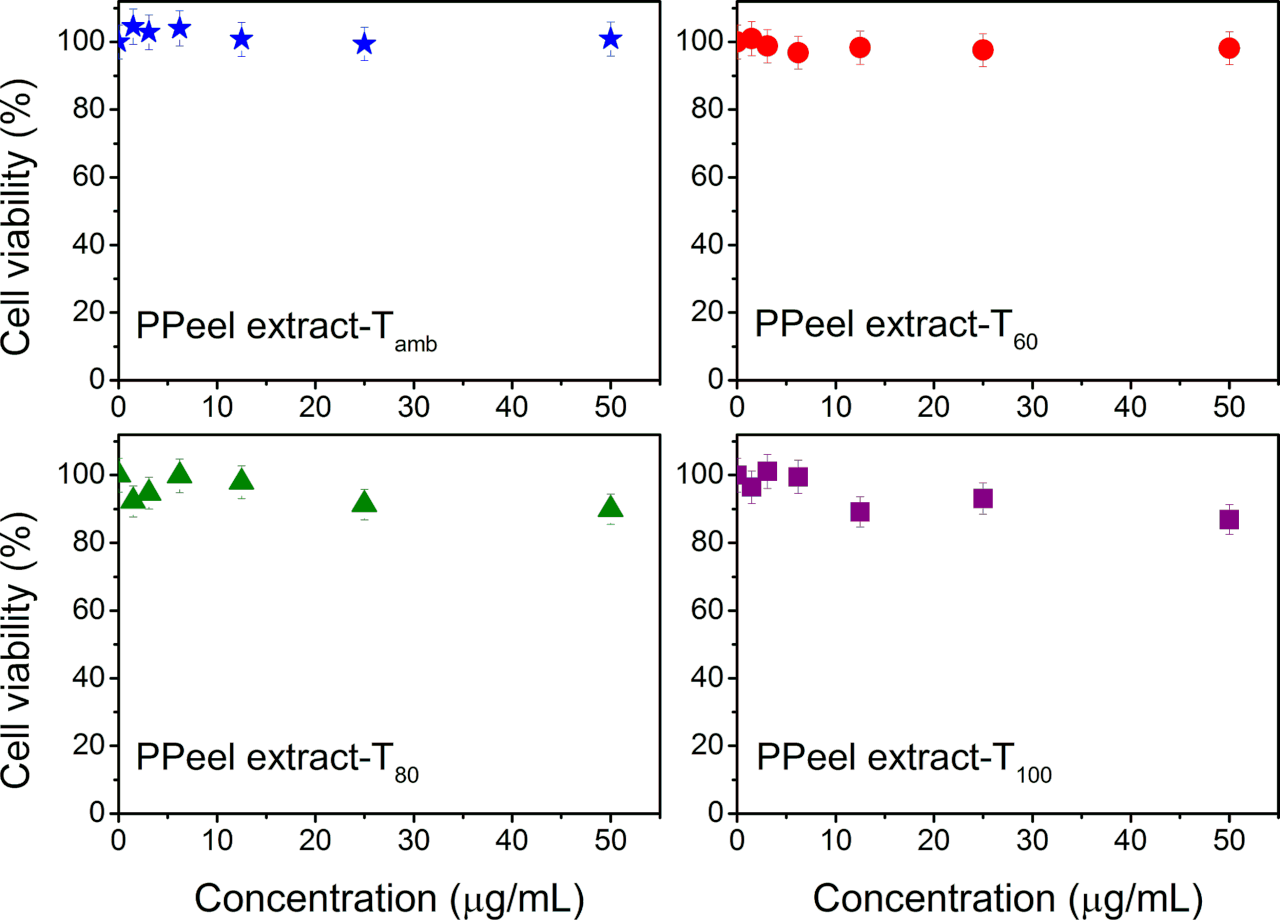
Cell viability assays on MCF-7 cells with pineapple peel extracts obtained at room temperature, 60, 80, and 100 °C.

In order to rule out whether the metabolites of the pineapple peel extract, necessary for the formation and stabilization of the nanoparticles, participate in the cytotoxic action, MCF-7 cell viability tests were performed on the extracts. The results are shown in Figure 11. In all cases, regardless of the temperature, there was no evidence that the extract had cytotoxic activity against MCF-7 cells. Therefore, the above cytotoxicity behavior can only be attributed to the Ag/AgCl nanoparticles obtained at different temperatures.

The results of the cytotoxic activity of AgNO_3_ tested both in peripheral blood mononuclear cells (PBMC) and in breast cancer cells (MCF-7) are shown in Table 3. The maximum concentration of AgNO_3_ tested was the one used to generate the Ag/AgCl nanoparticles. Even at the lowest concentrations, a significant decrease in cell viability was observed. The data indicated that AgNO_3_ had strong cytotoxic activity in both PBMC and MCF-7 cells; however, no cytotoxic selectivity was observed.

**Table 3:** Data on the cytotoxic activity of AgNO_3_ in peripheral blood mononuclear cells (PBMC) and in breast cancer cells (MCF-7).

AgNO_3_ (mM)	PBMC viability (%)	MCF-7 cells viability (%)

0	94.4 ± 11.6^a^	97.75 ± 12.12
0.031	29.1 ± 5.28^a^	24.54 ± 5.67
0.070	24.8 ± 1.84^a^	26.49 ± 2.31
0.150	27.2 ± 5.26^a^	28.20 ± 5.88
0.312	27.1 ± 1.3^b^	33.63 ± 5.82
0.620	27.8 ± 2.32^a^	22.01 ± 2.13
1.250	6.92 ± 0.62^a^	7.139 ± 0.44
2.500	6.86 ± 0.72^a^	7.080 ± 0.64
5.000	8.07 ± 1.28^a^	6.962 ± 0.20
10.000	7.55 ± 1.41^a^	7.021 ± 0.41

Experimental data points are presented as the mean ± SD of three independent experiments, with three replicates. Significant differences are indicated by ^a^*p* ≤ 0.0001 versus 0 mM and ^b^*p* ≤ 0.01 versus 0 mM.

## Conclusion

This study demonstrates the combined production of Ag and AgCl nanoparticles obtained through a green synthesis method. Pineapple peel was used for the synthesis method, where phenolic compounds were found, whose reducing capacity allowed for the formation of Ag/AgCl nanoparticles. Four biosynthesis temperatures were tested (i.e., room temperature, 60, 80, and 100 °C). The size, shape, and size distribution of the nanoparticles were affected by the temperature. Preferably, spherical nanoparticles with a size between 10 and 70 nm were obtained. It was noticeable that at 80 °C, triangular plates were also formed. The cytotoxic activity of the biosynthesized products against the MCF-7 breast cancer cell line was tested. The results showed a high cytotoxicity in these cells, up to 80% of those with the products obtained at temperatures of 60 and 80 °C. In contrast, these products showed a lower cytotoxic activity in PBMC healthy cells. Hence, it is reported for the first time that with this combined system of Ag/AgCl nanoparticles synthesized at a controlled temperature, a cytotoxic selectivity between cancer cells and healthy cells can be achieved.

## Experimental

### Materials

MD2 hybrid pineapples (family: Bromeliaceae, genus: *Ananas* Mill, 1754, species: *comosus* (L.) Merr., 1917) were obtained from crops in the Tuxtepec region of the state of Oaxaca, Mexico. Silver nitrate (CAS 7761-88-8, ACS reagent, purity ≥99.0%, purchased from Sigma-Aldrich Co) was used as a silver nanoparticle precursor.

### Aqueous extraction by infusion of pineapple peel

MD2 pineapples were peeled and used. The peel was cut into small pieces, and dried to a constant weight. Then, 100 g of dehydrated pineapple peel was weighed and put into a coffee filter. Subsequently, an infusion was made with 1 L of distilled water in a Oster^®^ brand coffee maker. The solution obtained was vacuum filtered and concentrated in a rotary evaporator at 50 °C, 250 rpm, and 42 mbar.

### Biosynthesis of Ag/AgCl nanoparticles

Quantification of phenolic and flavonoid compounds in the pineapple peel extract was performed before biosynthesis. Total phenolic content was performed using the method described by Singleton et al. [49], and the total flavonoid content was performed according to the method described by Dewanto et al. [50]. The biosynthesis of Ag/AgCl nanoparticles was carried out in a reflux system with a water bath at a controlled temperature. A proportion of 90% of AgNO_3_ salt (at a concentration of 10 mM) and 10% of pineapple peel extract (at a concentration of 10% w/v) was used. The synthesis was performed under constant stirring at 500 rpm for 2 h. The temperature was established as the study variable: room temperature, 60, 80, and 100 °C were the temperatures studied. Finally, the synthesized products were dried for 24 h for subsequent characterization. Some of the products of each reaction were kept at room temperature before drying to be characterized by UV–vis spectroscopy.

### Characterization

X-ray diffraction patterns were obtained in a Bruker AXS D8 Advance diffractometer, at 30 mA and 40 kV, with a Ni filter and a Cu Kα radiation generator. Diffraction patterns were acquired at a scan rate of 1 °/min from 10 to 90° in 2θ. Quantitative chemical analysis was performed on a JEOL JSM-7401F field-emission scanning electron microscope (FE-SEM) using EDX. An acceleration voltage of 15 kV and a working distance of 8 mm were used. The samples were precoated with Au/Pd for 10 seconds. Ultraviolet–visible spectroscopy was obtained using a Perkin Elmer Lambda 25 spectrophotometer, operating in the range of 350 to 700 nm. Fourier-transform infrared spectra were obtained with a Perkin Elmer Dynascan Spectrum 100 spectrometer, using an attenuated total reflectance (ATR) interferometer, operating in the range of 4000–600 cm^−1^. Transmission electron microscopy images were acquired on a JEOL 1010 microscope, with an accelerating voltage of 80 kV. For that, samples were pre-prepared in acetone and sonicated for 20 min, then dried at room temperature. Thermogravimetric analysis was performed on a Perkin Elmer STA 6000 simultaneous thermal analyzer, with a heating rate of 20 °C/min, under a nitrogen atmosphere, and a temperature range of 30–800 °C. To carry out XRD, EDX, FTIR, and TGA analyses, the pineapple peel extract and the reaction products were previously dried at 110 °C for 24 h.

### Cell culture

In a similar manner to [51], the MCF-7 human breast cell line was obtained from the American Type Culture Collection (ATCC®). This cell line was at passage 4 when used in the study and was routinely cultured on monolayers at 80% confluence in Dulbecco's modified Eagle's high glucose medium (DMEM), supplemented with 10% of fetal bovine serum, 100 U/mL of penicillin, 100 µg/mL of streptomycin, and 2 mM ʟ-glutamine. The cells were kept at 37 °C with saturated humidity and 5% CO_2_. The culture medium was removed to collect the human breast cancer cells, which were then washed with phosphate buffered saline (PBS). A cell dissociation solution made of trypsin-EDTA was added and incubated at 37 °C for 3 min in a humidified incubator with 5% CO_2_ to produce a cell suspension. Trypsinized cells were reseeded in fresh medium at 10^5^ cells/mL and incubated at 37 °C in a 5% CO_2_ humidified incubator. All reagents were purchased from Biowest, Riverside, USA.

In a similar manner to [52], peripheral blood mononuclear cells were obtained from blood samples of healthy adult human volunteers, using standard Histopaque 1077 (Sigma-Aldrich, St. Louis, MO. USA) techniques and density gradient centrifugation. PBMCs were maintained in Roswell Park Memorial Institute medium at pH 7.4, supplemented with 10% of fetal bovine serum, 100 U/mL of penicillin, 100 μg/mL of streptomycin, and 2 mM of ʟ-glutamine. All reagents were purchased from Biowest, Riverside, USA. PBMCs were kept at 37 ºC with saturated humidity and 5% CO_2_. All procedures performed followed the ethical standards of the institutional and/or national research committee and the 1964 Declaration of Helsinki. According to the Ethics Committee on Human Beings of the Universidad del Papaloapan (signed informed consent was not needed).

### Cell viability by MTT assay

The MTT assay is based on the ability of live cells to selectively reduce the yellow soluble salt MTT (3-(4,5-dimethylthiazol-2-yl)-2,5-diphenyl tetrazolium bromide, Sigma-Aldrich) to a purple/blue insoluble formazan crystal. For each sample, three independent experiments were performed in triplicates. Cell viability of control, Ag/AgCl-T_room_, Ag/AgCl-T_60,_ Ag/AgCl-T_80_, and Ag/AgCl-T_100_ samples were evaluated in 96-well flat-bottom culture plates (TPP). The MMT assay was performed on MCF-7 breast cancer cells and mononuclear cells. An amount of 2 × 10^4^ MCF-7 cells and 2 × 10^5^ PBMCs in 100 μL was seeded onto each well and incubated for 24 h at 37 °C, 5% CO_2_. After this time, and starting from a stock of 10 mg/mL prepared in PBS with serial dilutions, 0.05 mg/mL of the study samples was added. Subsequently, they were incubated at 37 °C and 5% CO_2_ for 24 h. Then, 10 µL of MTT solution prepared in PBS at a concentration of 5 mg/mL was added to MCF-7 cells, and an equal amount of MMT solution was added to monocytes. MCF-7 cells were incubated again for 4 h and mononuclear cells were incubated for 6 h, both at 37 °C and 5% CO_2_. The media were removed and 100 µL of dimethyl sulfoxide (DMSO) was added. The samples were then incubated at room temperature until the formazan crystals were dissolved. Finally, the absorbance at 570 nm was measured.
